# Acute Alcohol Effects on Response Inhibition Depend on Response Automatization, but not on GABA or Glutamate Levels in the ACC and Striatum

**DOI:** 10.3390/jcm9020481

**Published:** 2020-02-10

**Authors:** Wiebke Bensmann, Nicolas Zink, Annett Werner, Christian Beste, Ann-Kathrin Stock

**Affiliations:** 1 Cognitive Neurophysiology, Department of Child and Adolescent Psychiatry, Faculty of Medicine, TU Dresden, Fetscherstrasse 74, 01307 Dresden, Germany; Wiebke.Bensmann@ukdd.de (W.B.); Nicolas.Zink@ukdd.de (N.Z.); christian.beste@ukdd.de (C.B.); 2 Institute and Clinic for Diagnostic and Interventional Neuroradiology, TU Dresden, Fetscherstrasse 74, 01307 Dresden, Germany; Annett.Werner@ukdd.de

**Keywords:** alcohol, automatization, GABA, glutamate, response inhibition

## Abstract

Alcohol increases GABAergic signaling and decreases glutamatergic signaling in the brain. Variations in these neurotransmitter levels may modulate/predict executive functioning. Matching this, strong impairments of response inhibition are one of the most consistently reported cognitive/behavioral effects of acute alcohol intoxication. However, it has never been investigated whether baseline differences in these neurotransmitters allow to predict how much alcohol intoxication impairs response inhibition, and whether this is reflected in neurophysiological measures of cognitive control. We used MR spectroscopy to assess baseline (i.e., sober) GABA and glutamate levels in the anterior cingulate cortex (ACC) and striatum in *n* = 30 healthy young males, who were subsequently tested once sober and once intoxicated (1.01 permille). Inhibition was assessed with the sustained attention to response task (SART). This paradigm also allows to examine the effect of different degrees of response automatization, which is a known modulator for response inhibition, but does not seem to be substantially impaired during acute intoxication. As a neurophysiological correlate of response inhibition and control, we quantified EEG-derived theta band power and located its source using beamforming analyses. We found that alcohol-induced response inhibition deficits only occurred in the case of response automatization. This was reflected by decreased theta band activity in the left supplementary motor area (SMA), which may reflect modulations in the encoding of a surprise signal in response to inhibition cues. However, we did not find that differences in baseline (i.e., sober) GABA or glutamate levels significantly modulated differences in the size of alcohol-induced inhibition deficits.

## 1. Introduction

The aberrant consumption of alcohol is a significant cause of physical, mental, and social problems [1]. Acute alcohol intoxication and alcohol use disorder (AUD) strongly impair executive functions [2]. These impairments may contribute to maladaptive and dysfunctional behavior [3] that affects different aspects of life, including occupation [4].

Severe cognitive deficits are consistently found in the domain of inhibitory control, which is highly important to control (habitual) drinking, especially in the face of negative consequences [5]. While response inhibition becomes impaired by acute alcohol intoxication, it has been shown that automated processes, which also strongly modulate inhibitory control [6], seem to be largely spared from the detrimental effects of alcohol; even at high intoxication levels (≥1.2‰) [7,8,9]. Against this background, we hypothesized that the effect of acute alcohol intoxication on inhibitory control should depend on the degree of automatization. To investigate the role of response automatization for alcohol-induced response inhibition deficits, we asked healthy young males to perform three sustained attention to response task (SART) blocks in a sober and in an intoxicated state. Importantly, these blocks varied in the frequency of GO trials, which allows to examine the effect of different degrees of response automatization on inhibitory control processes [10,11,12,13]. With an increasing degree of automatization, more inhibitory control is required. Given that inhibitory control is impaired by alcohol intoxication [5], while automatization is not (to the same degree), we hypothesized that alcohol-induced inhibition deficits increase when the degree of response automatization increases. This should be reflected by stronger alcohol-induced decreases in NOGO accuracy in case of stronger automatization [6,14].

Regarding underlying neurophysiological processes, theta frequency oscillations play an important role [15,16,17,18,19]. Theta oscillations, which originate from medial frontal structures play a key role in cognitive control, especially in response inhibition [20,21,22,23,24] and are thought to reflect task-related response selection and control processes [18,25]. As medial frontal theta band activity is known to increase in many control situations [20], it can be hypothesized that an alcohol-induced performance decrease should be associated with reduced theta band activity. The neuroanatomical source of differences in theta band activity may be identified by means of beamforming [26,27,28]. The anterior cingulate cortex (ACC) and supplementary motor area (SMA) are involved in mediating automatic motor activation and the suppression of unwanted action plans [29]. Due to this special role, they should likely underlie alcohol-associated modulations of theta band activity.

However, it is important to consider that theta oscillations reflecting cognitive control are closely related to gamma-aminobutyric acid (GABA) and glutamate levels in the medial frontal cortex [30] and the striatum [19], which is anatomically well-connected to the medial frontal cortex [31]. Due to this close feed-forward connectivity, the functional contributions of these two brain regions to inhibition cannot be clearly separated. Moreover, the abundant expression of GABA and glutamatergic neurons in the striatum [32] is a major computational element during response selection [33,34,35]. In line with this, previous studies have suggested that GABAergic and glutamatergic signaling in the ACC and the striatum modulate impulsivity and inhibition [19,36,37]. This is of particular relevance in the context of alcohol intoxication effects because medial frontal and striatal regions are known to be modulated by alcohol [38,39] and this modulatory effect may be driven by alcohol-induced changes in GABAergic and glutamatergic signaling [40,41]. Specifically, alcohol increases GABAergic signaling [42,43,44,45,46,47,48] and decreases glutamatergic signaling [40] during acute intoxication. Moreover, it has been demonstrated that differences in baseline GABA levels in the ACC may explain differences in how young adults react to a binge-like alcohol intoxication in a clinical measures of brain functioning (e.g., black-outs), and how well frequent binge drinkers might perform on inhibition tasks while sober [49]. Importantly, these findings raise the question of whether baseline GABA levels in the ACC and striatum might also explain differences in acute cognitive alcohol effects, i.e., in how much response inhibition becomes compromised during an acute alcohol intoxication. Against this background, we set out to investigate whether inter-individual differences in baseline (i.e., sober) levels of striatal and ACC GABA and glutamate allowed predicting inter-individual differences in how strongly response inhibition becomes compromised by a standardized alcohol binge. In other words, we wanted to investigate whether sober GABA and glutamate levels in functionally relevant brain areas predict the size of detrimental alcohol effects on cognition and behavior. To examine this, we used magnetic resonance spectroscopy (MRS). With this method, it is possible to examine GABA and glutamate concentrations in special regions of interest and relate this structure-specific neurobiochemical data to EEG and behavioral correlates of inhibitory control.

In short, we hypothesized that the effect of acute alcohol intoxication on response inhibition depends on the degree of response automatization. As a consequence, we expected increased alcohol-induced impairments in inhibitory control with an increased degree of response automatization. We further hypothesized that these deficits might be caused by an alcohol-induced increase in GABA and decrease in glutamate levels in the striatum and ACC. If this was the case, inter-individual differences in striatal and ACC GABA and glutamate should correlate with the magnitude of the alcohol-induced inhibition deficit. Lastly, we examined whether the investigated effects were reflected in the theta band and associated activity modulations in medial frontal structures.

## 2. Experimental Section

### 2.1. Participants

A group of *n* = 39 healthy young male subjects between 18 and 30 years of age participated in the study. The sample size was based on an earlier study by Quetscher et al. [19], who tested a comparable number of participants for correlations between MRS and inhibition measures. All participants had been recruited using flyer and online ads at the local university (TU Dresden, Germany). Before admitting participants to the study, we checked all of the following inclusion criteria for each applicant: Participants needed to state that they were healthy and did not suffer from any psychiatric or neurological disorder or somatic diseases and did not take any prescribed medicine, especially drugs that might interfere with the function of the central nervous system, liver, kidneys, and gastrointestinal tract. Participants were further required to have normal or corrected-to-normal vision, and to be right-handed. Lastly, all participants needed to report a stable pattern of moderate, non-risky alcohol consumption habits. This was defined as Alcohol Use Disorders Identification Test (AUDIT) scores between 1 and 15 points [50], and reporting to binge drink (i.e., consume eight or more standard units of alcohol on a single occasion/ evening) no more than once a month and no less than once a year. Women were not permitted to participate in the study due to the potential risk of undetected pregnancy. Furthermore, hormonal changes in the menstrual cycle that could potentially have modulated and thereby confounded GABAergic neural transmission [51]. Before starting the experiment, all participants gave written informed consent. After the end of their study participation, the participants were reimbursed with 85€ or course credits. The study was conducted in accordance with the Declaration of Helsinki and approved by the ethics committee of the Faculty of Medicine of the TU Dresden (EK293082014).

### 2.2. Experimental Design and Alcohol Administration

**Figure 1:**
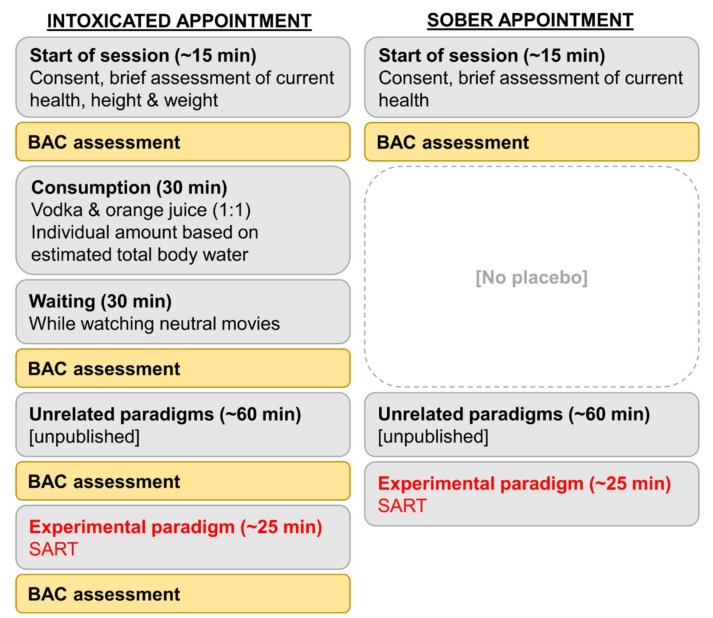
Illustration of the within-subject study design. Each subject was tested twice (i.e., once sober and once intoxicated). Between the sober and intoxicated appointment, there was always a time span of min. 48 h, and max. 7 days. The intoxication appointment started with brief assessment of current health and the parameters needed to calculate the individual amount of vodka (i.e., age, height, weight). Breath alcohol concentration (BrAC) was then assessed to confirm sobriety (i.e., 0.00‰) at the start of each appointment. Participants were asked to finish their drink within 30 min, and to wait another 30 min until the start of the experiment. Participants then spent one hour working on different, functionally unrelated tasks not reported in this paper. The task reported in this study took about 25 min to complete. BrAC was assessed immediately before and after each task during the intoxicated appointment. During the sober appointment, the participants were allowed to drink water, but we offered no placebo or waiting time, as previous piloting had clearly shown that participants could easily distinguish between placebo and vodka at the administered amount.

The study design and the administration of alcohol were identical to the protocol used in previous intoxication studies carried out by our group [7,9,52]. We used a cross-over design where each subject was tested twice (i.e., once sober and once intoxicated). The experimental procedure is illustrated in Figure 1. Between the sober and intoxicated appointment, there was always a time span of min. 48 h and max. 7 days. All participants were instructed to refrain from using caffeine, nicotine, guanine, and any other stimulant or sedative substances within the last 4 h before the start of each appointment, and to stop eating at least 3 h before the intoxication session. 

We administered individual amounts of alcohol, aiming for an average peak breath alcohol concentration (BrAC) of ~1.2‰. The amount of vodka (40 percent alcohol by volume) that each participant received was individually determined based on their estimated total body water (TBW). The Excel sheet which we used for the calculation of individual beverage amounts can be found in the Supplementary Materials. TBW was estimated with a version of the equation by Widmark [53] and Watson et al. [54]:

TBW=2.447−(0.09516×age)+(0.1074×height)+(0.3362×weight)

Each participant was given 1.98 g of alcohol per estimated liter of total body water. The alcohol was administered in the form of equal parts of vodka (40 vol. %) and orange juice. Both were measured and mixed at room temperature to minimize the risk of stomach aches. Irrespective of the individual amount that was served, all participants were asked to finish their drink within 30 min, and to wait another 30 min until the start of the experiment. In this time, the participants watched thee episodes of The Big Bang Theory in order to provide standardized entertainment that lacked extensive discourse on drinking and party-related topics and was intended to helped to prevent negative mood swings, as well as differential effects of the engagement in conversation with the experimenters [55,56]. After this mandatory waiting period, the participants spent one hour working on different, functionally unrelated tasks which are not reported in this article. As a consequence, the experiment started approximately 90 min after the end of the alcohol consumption. We used the “Alcotest 3000” breathalyzer (Drägerwerk, Lübeck, Germany) to assess BrAC at the beginning of each appointment (sober task performance, intoxicated task performance, MRS acquisition) and immediately before/after each task during the intoxicated appointment. Importantly, previous publications [47,57] have demonstrated that the obtained breath alcohol levels essentially yield the same results as a capillary gas chromatography-headspace analysis of venous blood. For the MRS acquisition, participants were invited to a third and separate sober scanning appointment, which also had to be within one week of testing, and no less than 48 h after the drinking appointment. MRS data collection lasted for approximately 1 h.

### 2.3. Experimental Paradigm

**Figure 2:**
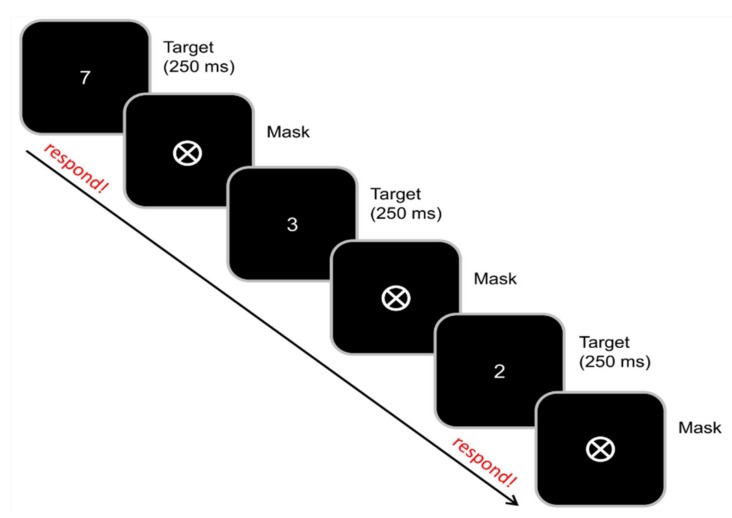
Schematic illustration of the experimental paradigm. The digits “1” to “9” were presented in a random order. The digit was followed by a mask (a circle with a cross inside). Participants were asked to react to any digit except “3”by pressing the space bar. Whenever the digit “3” was presented, the participants had to inhibit this response. The task was divided into three equally sized blocks. In the first block, there were eight times more GO trials than NOGO trials (one NOGO trial: eight GO trials), in the second block, the amount of GO trials and NOGO trials was equal (one NOGO trial: one GO trial), and in the third block, there were eight times more NOGO trials than GO trials (one GO trial: eight NOGO trials).

The sustained attention to response (SART) task allows to investigate the effect of different degrees of response automatization on inhibitory control processes. The task was originally introduced by Robertson et al. [58] and had already been used in previous, unrelated studies of our group [14,17]. The paradigm is illustrated in Figure 2.

All participants were seated at distance of about 57 cm from a 17 inch thin-film transistor (TFT) monitor and were asked to respond using a QWERTZ keyboard. In this GO/NOGO type task, the digits “1” to “9” were presented in a random order, with one digit shown in each trial. They were displayed in white font with randomly varied font size (67, 80, 107, or 120 pt). Each trial started with a 250 ms presentation of a single digit. The digit was followed by a mask (a circle with a cross inside). The inter-trial interval randomly varied between 1100 and 1600 ms. Participants were asked to rest their right index finger on the response button (space bar) and react to any digit except “3” by quickly pressing the button. Whenever the digit “3” was presented, the participants had to inhibit this response. The task was divided into three equally sized blocks.

In the first block, there were eight times more GO trials than NOGO trials (one NOGO trial: eight GO trials), so the participants likely had a strong automatic response tendency within this block. In the second block, the amount of GO trials and NOGO trials was equal (one NOGO trial: one GO trial), so the participants likely had a weaker response tendency than in the first block. In the third block, there were eight times more NOGO trials than GO trials (one GO trial: eight NOGO trials), so the participants likely had no pronounced automatic response tendency within this block. Each block consisted of 260 trials. All stimuli (digits) representing GO trials were presented equally frequently. Altogether, it took about 25 min to complete the task.

### 2.4. MRS Data Acquisition and Processing

**Figure 3:**
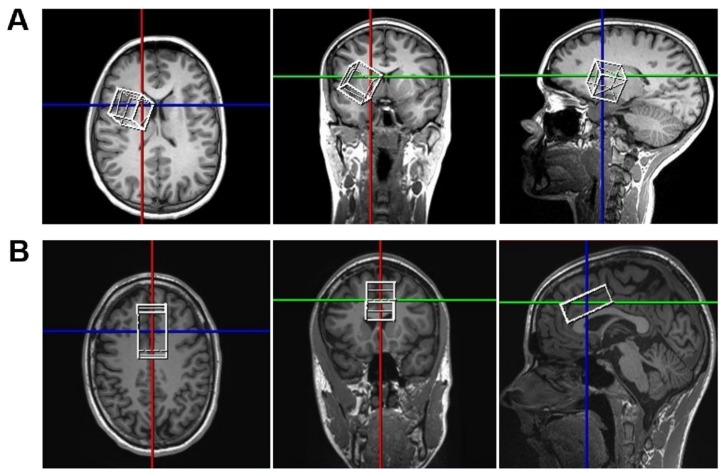
Positioning of the different volumes of interests (VOIs) used for the GABA+ spectroscopy. Figure part (**A**) shows an example for the positioning of the 30 × 30 × 30 mm VOI in the right striatum. In order to cover as much of the anterior and dorsal striatum as possible while avoiding the inclusion of ventricular CSF, the VOI was positioned in several consecutive steps. At starting point, the voxel was aligned along all three axes (colored lines) and positioned at the striatum (caudate and putamen), as checked in all three planes. Next, the voxel was rotated on the axial plane view (**left**) such that the anterior medial tip of the VOI pointed inwards/towards the sagittal line in order to cover as much as possible of the caput of the caudate nucleus. In the coronal view (**middle**), the lateral side of the VOI was then rotated upwards/in a dorsal direction such that the VOI aligned with the curvature of the lateral ventricle and covered as much as possible of the dorsal caudate nucleus. In the sagittal view (**right**), the VOI was then slightly rotated in the anterior direction in order to cover as much of the caudate and putamen as possible. Lastly, the positioning was double-checked in all three planes and, if necessary, its positioning was slightly adjusted/shifted along all three axes to make sure that the VOI did not cover any part of the lateral ventricles, or other larger CSF-containing structures. Figure part (**B**) shows an example for the positioning of the 20 (head-feet) × 60 mm (anterior-posterior) × 30 mm (left–right) VOI in the anterior cingulate cortex (ACC). In order to cover as much of the ACC as possible while avoiding the inclusion of ventricular CSF, the VOI was also positioned in consecutive steps. At the start, the voxel was aligned along all three axes (colored lines). In the sagittal view (**right**), the anterior part of the VOI was rotated ventrally and the VOI was shifted along the anterior–posterior and dorsal–ventral axes until the ventral border of the VOI aligned with the anterior upper border of the body of the corpus callosum and the anterior dorsal tip of the VOI reached the anterior border of the ACC. In the axial view (**left**), the VOI was shifted and rotated laterally until its sagittal center aligned with the midline of the brain. If necessary, the VOI was slightly rotated and shifted in the coronal view (**middle**) to make sure it did not contain any part of the lateral ventricles.

The concentrations of GABA+ in the striatum and the ACC were examined using 1H-MR-spectroscopy (MRS). MRI and MRS data were acquired using a 3T “Prisma” scanner (Siemens Healthineers, Erlangen, Germany) and a 32 channel (receive only) headcoil. Subsequent to the localizer, a high-resolution 3D T1-weighted sagittal Magnetization Prepared RApid Gradient Echo (MPRAGE) sequence (1 mm isovoxel) was measured and multiplanar reconstructed for exact voxel placements. Next, 30 × 30 × 30 mm voxels of interest (VOIs) were placed in the left as well as the right striatum to be able to average out laterality effects, and a 20 × 20 × 60 mms VOI was placed over the midline to cover both the left and right ACC. The positioning of the VOIs is shown and described in Figure 3. Of note, the ACC VOI covered large portions of the structure we wanted to assess and contained relatively small fractions of neighboring brain regions. In contrast to this, the striatal VOI also contained structures that were not directly relevant to our research question (compare Figure 3A). The main reason for this is that such a large voxel size is currently still required to obtain a good signal that allows for reliable quantification of GABA+ levels [59]. Additionally, the placement of the striatal VOI was optimized to cover as much as possible of the anterior and dorsal parts of the striatum, with the ventral and posterior striatum oftentimes not being (entirely) included in the VOI. This decision was based on the findings that within the striatum, the caudate is considered most functionally relevant for cognitive control processes, as it receives input from association areas. The putamen mainly receives motor and sensory input and is therefore considered to be more relevant for motor aspects of behavior. The ventral striatum receives input from limbic structures and is most closely associated with reward learning, which was not assessed in our study [60,61,62]. Furthermore, the VOI placement was in line with that of previous studies which have successfully demonstrated the functional relevance of striatal GABA for response inhibition [19,63].

To improve spectral resolution, a special shim adjustment based on Gradient Echo (GRE) Double Acquisition (Work In Progress, Siemens) was performed, followed by manual shimming, to achieve a full width at half maximum (FWHM) value below 15 Hz. MRS data were acquired using the Center for Magnetic Resonance Research *Mescher-Garwood* point-resolved spectroscopy (CMRR-MEGAPRESS) sequence (TE/TR = 68/2000 ms, edit ON acquisitions = 128, edit OFF acquisitions = 128) developed by Edward J. Auerbach and Małgorzata Marjańska [64,65] and provided by the University of Minnesota under a C2P license agreement. In addition, short-echo-time point resolved spectroscopy (PRESS) spectra (echo time TE/repetition time TR: 30/2000 ms) with (number of scans NS: 256) and without water suppression (NS: 16, for eddy current corrections) were performed for a more reliable estimation of other major brain metabolites (glutamate, or “Glu”). The LCModel (v6.3-1H) [66], which fits the in vivo MR spectra as a linear combination of single metabolite “basis spectra”, was used to quantify the spectra. TE 68 basis sets were delivered by the group of Ulrike Dydak [67] and generated from density matrix simulations of the sequence, using published (updated) values for chemical shifts and J- GABA coupling constants from Kaiser et al. [68,69,70] with an exact treatment of metabolite evolution during the two frequency-selective MEGA inversion pulses. Difference basis spectra were obtained by averaging the simulated metabolite response to selective inversion at 1.9 and 7.5 ppm. Based on the “edit off” spectra from the identical measurement, reference values for GABA+ of creatine (Cr) or N-acetylaspartate (NAA) were estimated (using a specially designed LC-model dataset [67]). Reference values (Cr) for Glu were obtained from the PRESS-sequence (TE/TR: 30 ms/2000 ms) using the corresponding basis data set. For data quality reasons concerning the subsequent quantification of MRS spectra, only spectra of adequate shim quality (≤20 Hz) were used. Prior to the correlation analyses integrating the MRS data with the behavioural data, the concentrations of the different metabolites were averaged across the VOI placed in the left and the right striatum. This was not the case for the ACC, since the voxel was placed at the midline and already covered the left and right ACC. MRS testing was always conducted prior to the experimental paradigms, because GABA+ levels are known to be subject to circadian changes (higher concentrations at night) [71]. This way, MR spectroscopy measurements were conducted at the same time in the afternoon throughout the whole study.

### 2.5. EEG Recording and Time–Frequency Analyses

EEG data recording followed the standard protocol applied by our group. We used an EEG cap with 60 Ag-AgCl electrodes at standard equidistant scalp positions. Electrode impedances were kept below 5 kΩ, and the data was recorded against a reference electrode at position Fpz, using a QuickAmp amplifier (Brain Products GmbH, Gilching, Germany). The data were initially recorded at a sampling rate of 500 Hz. Brain Vision Analyzer 2.1. software (Brain Products GmbH, Gilching, Germany) was used for offline data pre-processing and EEG data analyses. During this process, the data were downsampled to 256 Hz and a band-pass filter (infinite impulse response filter from 0.5 to 20 Hz with a slope of 48 db/oct) was applied. Technical or irregular movement artifacts were then eliminated with the help of a manual raw data inspection. An independent component analysis (ICA; infomax algorithm) was then applied to remove periodically recurring artifacts like eye movements or pulse artifacts. Lastly, a second manual raw data inspection was conducted to remove any residual artifacts that might have prevailed in the EEG data up to this point. In the next step, EEG data were segmented in a target-locked fashion. Each segment started 2000 ms before and ended 2000 ms after target stimulus onset (set to time point zero) to ensure having enough full cycles at theta frequency. Only correctly answered trials were included in the analyses. Additionally, an automated artifact rejection procedure was applied. Here, we excluded segments with amplitudes below −100 and above 100 μV. The maximally allowed value difference in a 200 ms interval was set to 200 μV and the lowest acceptable amplitude difference in a 100 ms time span was set to 0.5 μV. Furthermore, we eliminated the reference potential by conducting a current source density (CSD) transformation. In addition to serving as a spatial filter, CSD transformation may improve the identification of electrodes that best reflect ERP peaks and associated cognitive processes [72,73]. A baseline correction was applied to the interval of −500 to −100 ms before target onset. 

Time–frequency (TF) decomposition was achieved using a continuous wavelet transform (CWT) that applies Morlet wavelets to different frequencies in the time domain. For this study, the TF decomposition was performed in 0.5 Hz intervals from 0.5 to 20 Hz. The Morlet parameter, which is used to plot the time–frequency (*σf* = width of Gaussian shape in the frequency domain; *f*0 = central frequency) was set to *f*0/*σf* = 5.5. To calculate the total power, TF decomposition was separately applied to the single-trial data of each experimental condition. For the power band quantification, we focused on central theta oscillations, which are known to play an important role in executive control functions [20,74]. Electrodes were chosen based on cluster-based permutation tests from the fieldtrip toolbox [75] based on the theta power scalp topographies, following the procedure described by Oostenveld et al. [76]. We ultimately used the a frequency window of 4–6 Hz and a time window of 300–350 ms after target onset to determine the mean power, because the power was maximal at this frequency and time window. To compute the dependent samples *t* statistics, samples were considered as candidate members of a cluster of samples if their *t* value exceeded 0.05. By means of the Monte Carlo method, the reference distribution of the permutation test was approximated using 2000 random draws. A cluster was considered significant if its *p* value was below the critical alpha level of *p* < 0.025. Within the theta band, we quantified the power at a frequency of 5 Hz in the time frame from 300 ms to 350 ms at electrode Cz, where the theta power reached its maximum.

### 2.6. Beamforming

Finally, beamforming analyses were used to identify the neuroanatomical structures underlying differences of interest in the investigated total theta band power range. Because the CSD transformation and the beamformer both work as spatial filters [72], we applied the wavelet transformation procedure without prior CSD transformation, so that the segmented data retained its average reference [77]. In the spectral analysis, the power and the cross spectral density matrix were computed using a multitaper frequency transformation. Matching the TF quantification described above, theta band core frequency was set to 5 Hz with a smoothing window of 1.00 Hz, but instead of the time window from 300 to 350 ms used in the initial TF quantification, we used a time interval from 0 ms to 600 ms after target onset. The main reason for doing so was that this time window ensurs that at least three cycles of oscillations could be used for the beamforming analysis, which is necessary to ensure sufficiently reliable beamforming sources. A Dynamic Imaging of Coherent Sources (DICS) beamformer was used to reconstruct the cortical sources of the oscillatory theta band activity [77]. Of note, this linear beamforming approach has already been successfully used to reconstruct frequency-specific sources in several EEG and MEG studies [6,17,27,28,78]. The beamformer-based source reconstruction relies on a spatially adaptive filter that is subject to the unit-gain constraint. The beamformer filter specifications allow an estimation of the amount of activity at any given location in the brain, while maximally suppressing the activity of other locations. DICS beamforming was implemented using the “Fieldtrip” Matlab toolbox [76], which includes a Montreal Neurological Institute (MNI) brain-template-based forward model that has been described in detail by Oostenvend et al. [76]. EEG electrodes were realigned to this head model before a leadfield matrix was computed by partitioning the forward model’s brain volume into a grid with 6 mm resolution. Consecutively, the leadfield matrix was calculated for each grid point. A common spatial filter based on all conditions with the regularization parameter set to 5% was separately applied to each condition to estimate the power of the sources. Importantly, the DICS beamformer was only applied to the TF intervals that had yielded significant difference effects in the previous wavelet analyses (see Section 3.4 for details). The source-power estimates for each condition were contrasted by computing the ratio between conditions normalized by the sum of conditions:

$$P_{ratio}=\frac{P_{cond1}-P_{cond2}}{P_{cond1}+P_{cond2}}$$

where *P_cond_*_1_ and *P_cond_*_2_ are the contrasted conditions. This approach cancels out a possible noise bias and reduces the effect of outliers by assuming that the noise is distributed equally in both conditions. These theta source power estimates are given in MNI coordinates [76,79]. The procedure we used is comparable to other studies conducted by our group [78,80,81,82].

### 2.7. Statistics

Behavioral and electrophysiological data were analyzed using repeated measures analyses of variance (ANOVAs) comprising the within-subject factors “intoxication status” (sober vs. intoxicated), “automatization block” (block 1 vs. block 2 vs. block 3), and “condition” (GO vs. NOGO). All performed tests were Greenhouse–Geisser-corrected, whenever necessary. In the results section, the mean and standard errors of the mean (SEM) are reported as a measure of variance. MRS data were integrated with the behavioral and neurophysiological data by means of correlation [19,63,83,84]. For this, the individual GABA and glutamate concentrations in the ACC and striatum at baseline (i.e., in the sober state) were used to predict the behavioral differences between sober and intoxicated (i.e., sober–intoxicated) appointments for each combination of experimental conditions. As there was no reason to assume lateralization effects, GABA and glutamate concentrations in the right and left striatum were averaged across both sides.

## 3. Results

### 3.1. Exclusion of Participants and Sample Characterization

After collecting the data, nine participants from the initial sample of *n* = 39 participants were excluded for the following reasons: N = 3 participants had to discontinue the experiment before the paradigm could be run due to vomiting after alcohol administration (of note, those cases are not included in the uploaded dataset). N = 2 participants had to be excluded due to technical issues (i.e., EEG was not recorded correctly). N = 2 participants were excluded for slow responding (mean RT of more than 600 ms during the sober appointment). N = 1 participant stopped pressing the response button after a while on one of the appointments. This was most likely due to an accidental shift on the keyboard resulting in responding with an incorrect button (which was not recorded). Lastly, *n* = 1 participant secretly watched movies on his smartphone during the experiment (all participants had been instructed to shut off their smartphones and put them away while the experimenters were watching to avoid potential interference with EEG recording, but this participant seems to have carried a second device that we were initially not aware of). On average, the *n* = 30 participants who remained in the sample were 23.43 ± 0.717 years old (range 18–30), had a height of 181.6 ± 1.3 cm (range 169–195), a weight of 76.8 ± 1.9 kg (range 56–100), and an AUDIT score of 6.8 ± 0.6 (range 3–14).

### 3.2. Acute Intoxication

N = 14 participants received alcohol on the first of their two study appointments, while the remaining *n* = 16 participants received alcohol on their second appointment. Based on the estimated total body water in liters, the participants received an average amount of 281.63 ± 4.22 mL vodka, or 90.12 ± 1.35 gr alcohol. Immediately before performing the experimental paradigm (i.e., approx. 90 min after finishing their drink), the participants had a mean BrAC of 1.01‰ ± 0.02. After completing the paradigm, their mean BrAC was 0.93‰ ± 0.03.

### 3.3. Behavioral Data

**Figure 4:**
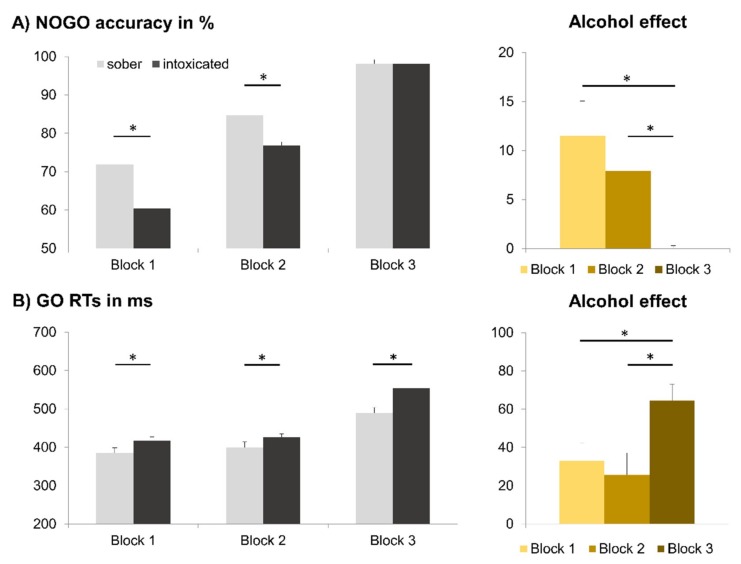
Behavioral data. (**A**) On the left, accuracy (mean percentage of correct responses) in NOGO trials is shown for the different automatization blocks (**left**), while the alcohol effect (i.e., sober minus intoxicated) is shown on the right. (**B**) On the left, GO trial reaction time (RT) data (mean reaction times) are shown for the different automatization blocks, while the alcohol effect (i.e., intoxicated minus sober) is shown on the right. Significant results (*p* ≤ 0.05) are denoted with an asterisk. Error bars show the SEM as a measure of variability.

The analysis of accuracy (percentage of hits) revealed a main effect of intoxication status (*F*(1, 29) = 24.246, *p* < 0.001, $\eta_{p}^{2}$ = 0.455), with fewer hits in the intoxicated state (87.73% ± 1.28) than in the sober state (91.74% ± 0.92). Moreover, there was a significant main effect of condition (*F*(1, 29) = 111.321, *p* < 0.001, $\eta_{p}^{2}$ = 0.793), with a higher number of hits for GO trials (97.79% ± 0.51) than for NOGO trials (81.68% ± 1.74). There was also a significant main effect of automatization block (*F*(1.381,40.048) = 92.065, *p* < 0.001, $\eta_{p}^{2}$ = 0.760). Post-hoc *t*-tests showed significant differences for all possible contrasts (all *t* ≥ 7.455; *p* ≤ 0.001), with a higher number of hits in block 3 (98.12% ± 0.40) than in block 2 (89.38% ± 1.18) or in block 1 (81.71% ± 1.76). Furthermore, there were significant interactions of intoxication status × condition (*F*(1, 29) = 7.848, *p* = 0.009, $\eta_{p}^{2}$ = 0.213), intoxication status × automatization block (*F*(1.517, 44.006) = 7.656, *p* = 0.003, $\eta_{p}^{2}$ = 0.209), and condition × block (*F*(1.397, 40.518) = 85.180, *p* < 0.001, $\eta_{p}^{2}$ = 0.746). Importantly, there was also an interaction of intoxication status × condition × automatization block (*F*(1.340, 39.853) = 6.829, *p* = 0.007, $\eta_{p}^{2}$ = 0.191). To analyze this interaction, we conducted separate analyses for GO trials and NOGO trials. In NOGO trials, there were main effects of intoxication status (*F*(1, 29) = 16.183, *p* < 0.001, $\eta_{p}^{2}$ = 0.358; intoxicated state = 78.45% ± 2.21; sober state = 84.91% ± 1.58) and automatization block (*F*(1.373,39.813) = 93.875, *p* < 0.001, $\eta_{p}^{2}$ = 0.764; block 1 = 66.11% ± 3.19; block 2 = 80.73% ± 2.08; block 3 = 98.20% ± 0.30), as well as an interaction of intoxication status × automatization block (*F*(1.366, 39.624) = 8.308, *p* = 0.003, $\eta_{p}^{2}$ = 0.223) (see Figure 4). Post-hoc testing revealed a significant alcohol effect in block 1 (*t*(29) = 3.211; *p* = 0.003; intoxicated = 60.37% ± 4.11; sober = 71.85% ± 3.14) and in block 2 (*t*(29) = 4.430; *p* < 0.001; intoxicated = 76.77% ± 2.67; sober = 84.69% ± 1.78). However, no such detrimental alcohol effect could be observed in block 3 (*t*(29) < 0.001; *p* > 0.999). Moreover, the intoxication effect (sober minus intoxicated) was significantly smaller in block 3 (<0.01% ± 0.30) than in either block 1 (11.48% ± 3.58) (*t*(29) = 3.347; *p* = 0.002) or block 2 (7.92% ± 1.79) (*t*(29) = 4.816; *p* < 0.001). The alcohol effect size did not differ between block 1 and block 2 (*t*(29) = 1.102; *p* < 0.280). In GO trials, there was only a main effect of intoxication status (*F*(1, 29) = 8.924, *p* = 0.006, $\eta_{p}^{2}$ = 0.235), but no such interaction. All other main and interaction effects were not significant (all *F* ≤ 1.099; *p* ≥ 0.332). In short, we found detrimental effects of alcohol on response inhibition to be present in case of response automatization, and to be absent when there was no response automatization. But while the intoxication effect appeared bigger in case of stronger automatization (block 1 vs. block 2), this effect did not reach significance.

The repeated measures ANOVA for RTs in correct GO trials only showed a main effect of intoxication status (*F*(1, 29) = 32.164, *p* < 0.001, $\eta_{p}^{2}$ = 0.526), as responses were slower in the intoxicated state (465.8 ms ± 12.1) than in the sober state (424.8 ms ± 11.4). There was also a main effect of automatization block (*F*(1.412, 40.952) = 105.999, *p* < 0.001, $\eta_{p}^{2}$ = 0.785). Post-hoc *t*-tests showed significant differences for all possible contrasts (all *t* ≥ 2.099; *p* ≤ 0.045), with slower responses in block 3 (522.1 ms ± 8.1), than in block 2 (412.7 ms ± 14.1) or in block 1 (401.1 ms ± 14.0). Moreover, there was a significant interaction of intoxication status × automatization block (*F*(1.890, 54.823) = 6.171, *p* < 0.004, $\eta_{p}^{2}$ = 0.175) (see Figure 4). Post-hoc *t*-tests showed significant differences between the intoxicated and the sober appointment for all three blocks (all *t* ≥ 2.220; *p* ≤ 0.034). Most importantly, the alcohol effect (intoxicated minus sober) was significantly larger in block 3 (64.5 ms ± 8.4) than in block 1 (32.8 ms ± 9.6) (*t*(29) = 2.583; *p* = 0.015) or block 2 (25.5 ms ± 11.5) (*t*(29) = 3.074; *p* = 0.005). The alcohol effect size did not differ between block 1 and block 2 (*t*(29) = 0.707; *p* > 0.485).

### 3.4. Neurophysiological Data

**Figure 5:**
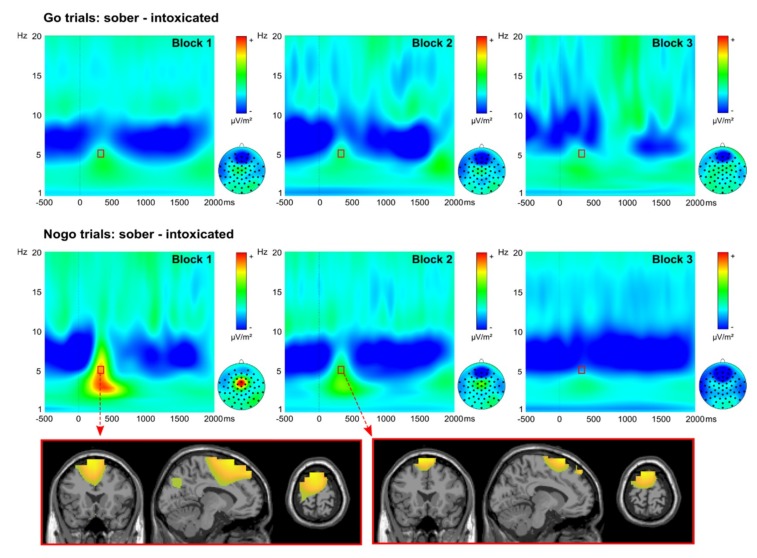
Alcohol-induced reductions in inhibition-associated theta power. The graphs show the time-frequency (TF) decomposition of the alcohol effect, i.e., the difference between the sober and intoxicated appointment. The alcohol effect of block 1 is shown in the left graph, the block 2 alcohol effect is shown in the middle graph, and the block 3 alcohol effect is shown in the right graph. 5 Hz theta band power was quantified at electrode Cz within the time interval from 300 to 350 ms (highlighted with rectangular boxes in each TF plot). The x-axis of each TF plot denotes the time in milliseconds (target stimulus onset at zero), while the y-axis denotes frequency and the color coding denotes power. Scalp topography plots are given for every condition at the same frequency and time interval. In GO trials (first row) the three blocks showed no significant theta differences. In NOGO trials (second row) blocks 1 and 2 showed significant theta differences (i.e., smaller theta band power at 5 Hz in the intoxicated state than in the sober state). The sources of those differences are illustrated in the maps below, where yellow color denotes significantly greater activation in the left SMA during sobriety, as compared to intoxication.

Wavelet plots illustrating the target-locked time–frequency decomposition of alcohol effects on the EEG signal are given in Figure 5.

We quantified and analyzed theta frequency band power at 5 Hz and at electrode Cz in a time frame from 300 to 350 ms. There was a main effect of intoxication status (*F*(1,29) = 6.072, *p* = 0.020, $\eta_{p}^{2}$ = 0.173), with stronger theta band activity in the sober state (237.29 μV/m^2^ ± 26.67) than in the intoxicated state (194.56 μV/m^2^ ± 16.93). Furthermore, we found a significant main effect of condition (*F*(1,29) = 29.164, *p* < 0.001, $\eta_{p}^{2}$ = 0.501), with a stronger theta band activity for NOGO trials (236.96 μV/m^2^ ± 23.16) than for GO trials (194.89 μV/m^2^ ± 18.47). There was also a significant main effect of automatization block (*F*(1.334,38.690) = 31.631, *p* < 0.001, $\eta_{p}^{2}$ = 0.522). Post-hoc *t*-tests showed significant differences for all possible contrasts (all t ≥ 3.742; *p* ≤ 0.001), with a lower theta band activity in block 3 (173.04 μV/m^2^ ± 16.32), than in block 2 (224.54 μV/m^2^ ± 22.15) or in block 1 (250.20 μV/m^2^ ± 24.73). Additionally, there were significant interactions of intoxication status × condition (*F*(1, 29) = 6.608, *p* = 0.016, $\eta_{p}^{2}$ = 0.186), intoxication status × automatization block (*F*(1.341, 38.899) = 7.711, *p* = 0.004, $\eta_{p}^{2}$ = 0.210) and condition × automatization block (*F*(1.534,44.488) = 27.977, *p* < 0.001, $\eta_{p}^{2}$ = 0.491). Importantly, there was also an interaction of intoxication status × condition × automatization block (*F*(1.740, 50.467) = 8.544, *p* = 0.001, $\eta_{p}^{2}$ = 0.228) (see Figure 5). To analyze this interaction, we conducted separate analyses for GO trials and NOGO trials. In NOGO trials, there were main effects of intoxication status (*F*(1,29) = 6.871, *p* = 0.014, $\eta_{p}^{2}$ = 0.192; intoxicated state = 204.97 μV/m^2^ ± 17.96; sober state = 268.96 μV/m^2^ ± 32.38) and automatization block (*F*(1.272, 36.883) = 35.768, *p* < 0.001, $\eta_{p}^{2}$ = 0.552; block 1 = 317.38 μV/m^2^ ± 34.39; block 2 = 243.85 μV/m^2^ ± 25.56; block 3 = 149.66 μV/m^2^ ± 12.98), as well as an interaction of intoxication status × automatization block (*F*(1.254, 36.356) = 10.242, *p* = 0.002, $\eta_{p}^{2}$ = 0.261). Post-hoc testing showed significant alcohol effects in block 1 (*t*(29) = 2.958; *p* = 0.006; sober = 387.80 μV/m^2^ ± 54.94; intoxicated = 246.96 μV/m^2^ ± 21.93) and in block 2 (*t*(29) = 2.649; *p* = 0.013; sober = 274.92 μV/m^2^ ± 32.96; intoxicated = 212.77 μV/m^2^ ± 22.25), but not in block 3 (*t*(29) = 1.118; *p* = 0.273). Additionally, the intoxication effect (sober minus intoxicated) significantly differed between all automatization blocks (all *t* ≥ 2.356; *p* ≤ 0.025), with the smallest effect in block 3 (-11.03 μV/m^2^ ± 9.86), a medium effect in block 2 (62.16 μV/m^2^ ± 23.46), and the largest alcohol effect in block 1 (140.84 μV/m^2^ ± 47.61). Beamforming analyses revealed that the alcohol-induced decrease in theta band activity in block 1 and block 2 was related to a stronger activation of the left supplementary motor area (SMA peak MNI coordinates in mm: 41/73/72) in the sober state (as compared to the intoxicated state) in both automatization blocks (see Figure 5). In the separate post-hoc analysis of GO trials, there were no such significant effects (all *F* ≤ 2.678; *p* ≥ 0.080). 

### 3.5. MRS Measures

In order to investigate whether any of the MRS-assessed transmitter levels correlated with the magnitude of alcohol-induced cognitive deficits, we performed correlation analyses. In order to reduce the chances of identifying false positive effects or mere epiphenomena, we only entered behavioral difference values (i.e., sober minus intoxicated) as behavioral measures of inhibition that had demonstrated to be significantly impaired by alcohol intoxication (i.e., NOGO trial accuracy in blocks 1 and 2). Importantly, none of these behavioral measures correlated significantly with any of the MRS-assessed GABA levels (all *p* ≥ 0.333), glutamate levels (all *p* ≥ 0.075), or the GABA/glutamate ratio (all *p* ≥ 0.351). Given that none of the correlations between neurotransmitter levels and behavioral measures reached significance, we decided to refrain from correlating neurophysiological measures with MRS data, as potential results could only have demonstrated behaviorally irrelevant epiphenomena. 

### 3.6. Summary of Main Results

Taken together, the data confirmed our main hypothesis on the role of automatization, as a detrimental effect of alcohol on inhibition could be found in case of response automatization (blocks 1 and 2), but was absent when there was no response automatization (block 3). This alcohol-induced inhibition impairment was reflected by decreased theta band activity in the left SMA, which is functionally relevant for the execution of the right hand GO responses. Importantly, we could exclude a speed/accuracy tradeoff, as the intoxicated participants did not only commit more false alarms, but also responded more slowly when intoxicated. However, neither baseline GABA levels nor baseline glutamate levels in the striatum and ACC predicted behaviorally relevant alcohol effects on inhibition, as we found no significant correlations for blocks 1 and 2.

## 4. Discussion

Acute alcohol intoxication is known to cause physical and mental harm. Drunk individuals commonly show reduced inhibitory control [5,85], whereas automated processes are assumed to be largely spared from the detrimental effects of alcohol [7,8,9]. Given that inhibitory control performance is strongly modulated by the degree of automatization of the very responses we are trying to control [17], we hypothesized that the effect of acute alcohol intoxication on response inhibition depends on the degree of underlying response automatization. On the neurophysiological level, we expected this relative and absolute lack of control to be associated with decreased theta band power, which originates in medial frontal structures and provides a neurophysiological correlate of the involved cognitive control processes [20,86]. GABAergic and glutamatergic signaling in fronto-striatal loops are known to play an important functional role for theta-associated inhibitory control processes [20,21,22,23,24], and they are modulated by the acute effects of alcohol [38,39,40,41]. Given that inter-individual differences in sober baseline levels of these transmitters have been suggested to predict how young adults respond to alcohol [49], we had hypothesized that inter-individual differences in baseline (i.e., sober) levels of striatal and ACC GABA and glutamate should allow to predict inter-individual differences in how strongly response inhibition becomes compromised during a standardized alcohol binge. 

To investigate this, we applied a task which allowed to examine the effect of different degrees of response automatization on inhibitory control processes. Reproducing previous publications, we found that an acute, binge-like alcohol intoxication of 1.01‰ significantly impaired (response) inhibition [8,9]. More importantly, however, we found that the detrimental effects of alcohol on inhibition were only found in the blocks/conditions that fostered response automatization (i.e., blocks 1 and 2), and were entirely absent when there was no task-relevant response automatization at all (block 3). The most likely reason for the observed absence of alcohol-induced inhibition failures is that a lower degree, or absence, of automatization reduced the demands on response inhibition. Therefore, it should have been much less difficult for participants to refrain from responding even in case of reduced control capacities [18,85]. In other words, intoxicated individuals do not require any noteworthy inhibitory skills to refrain from responding when no task-relevant automatic response is generated in the first place. As a consequence, they show no inhibition deficits in the absence of response automatization (as observed in block 3). As the intoxicated participants did not only commit more false alarms, but also responded more slowly, we could furthermore exclude a speed/accuracy tradeoff as an alternative explanation. However, we could not confirm our hypothesis that alcohol-induced inhibition deficits increase when the degree of response automatization increases. While alcohol-induced decreases in NOGO accuracy appeared to be a little larger in case of stronger automatization on the descriptive level (see Figure 4), the data unfortunately did not allow for the rejection of the null hypothesis.

Matching the behavioral results of impaired inhibitory control, we found alcohol-induced reductions in theta band power on the neurophysiological level. Further matching the modulation by response automatization, detrimental effects of alcohol on theta band power were evident when responses were automatized, and were absent when there was no response automatization. Theta band activity is important for cognitive control, especially for response inhibition [20,21,22,23,24], and may reflect task-related response selection and control processes [18,25]. Moreover, earlier studies have already demonstrated theta oscillations to be modulated by alcohol [87,88]. Our results therefore suggest that response inhibition is poorer under an acute influence of alcohol due to decreased cognitive control processes [20]. Corroborating this interpretation, the beamforming analysis showed that theta band modulations were associated with the left SMA. Like the ACC, this structure is well-known to be modulated by alcohol. Medial frontal theta oscillations originating in these mid-frontal brain regions have been shown to reflect cognitive control processes, including response inhibition [21,89,90,91]. The SMA is considered to play a key role in stopping a response [29]. Theta band activity in this structure likely reflects the coding of a “surprise signal” in response to a stopping cue, which leads to task-specific adjustments in cognitive control [20]. In case (a high degree of) response automatization results in particularly error-prone response inhibition, the “surprise” is coded by intensified theta band activity [25], which may help to inhibit further automatic responding [25]. Taken together, this suggests that the alcohol-induced decrease in SMA-related theta band power, suppresses the “surprise” signal generated in the ACC, which ultimately reduces the ability to reduce automatic responding when necessary [6,17]. Contrary to our expectations, we did not find that inter-individual differences in GABA and glutamate within fronto-striatal loops predicted the magnitude of alcohol-induced inhibition deficits. Response inhibition strongly depends on ACC and striatal information processing [92], which are both also known to be modulated by alcohol [38,39]. Additionally, these structures are affected by the alcohol-susceptible neurotransmitters GABA and glutamate [2,5,93]. Silveri et al. [49] had previously reported that ACC GABA levels allowed prediction of differences in how young adults react to a binge-like alcohol intoxication on a clinical symptom level (e.g., black out frequency). In contrast to this clinical measure of brain functioning, we investigated cognitive measures of brain functioning during intoxication. While it should be mentioned that Silveri et al. [49] also reported that ACC GABA levels were predictive of response inhibition in binge drinkers while sober, they only observed this in a rather small sub-sample of *n* = 14 binge drinkers and did not find this in non-binge drinkers, or investigate the acute effects of alcohol. While this requires further replication, it is hence possible that even though fronto-striatal GABA and glutamate play a key role in cognitive functioning, as well as in mediating the cognitive effects of alcohol intoxication, transmitter levels assessed at a sober baseline do not allow prediction of the degree of impairment caused by a standardized binge.

Most importantly, however, it should be stressed that this lack of effect does not in any ways imply that response inhibition deficits are not partly, or even mainly, caused by alcohol-induced changes in fronto-striatal GABA and glutamate signaling. It only states that it likely not possible to predict the degree of alcohol-induced reductions in response inhibition on the basis of transmitter levels at a sober baseline measurement. In this context, it also needs to be mentioned that while the ACC VOI barely covered any other brain structures apart from the region we intended to assess, the 3 × 3 × 3 cm VOI used for striatal GABA/glutamate quantification inevitably also contained structures that were not closely related to our research question. Indeed, such a large voxel size is currently required to obtain a good signal that allows for reliable quantification of GABA+ levels [59], and comparable striatal VOI placement has successfully applied in previous studies demonstrating the functional relevant of striatal GABA for response inhibition [19,63]. Still, we can of course not exclude the possibility that the obtained GABA and gluatmate levels were biased by neurotransmitter concentrations in other regions within the voxel, such as the insula. Hence, we cannot entirely exclude the possibility that striatal GABA effects might have been “masked” by varying concentrations in adjacent brain areas. Lastly, we had to exclude *n* = 9 participants from the initial sample because of various reasons. Therefore, the current study employed a relatively small sample size. As a result, it will also be important to replicate these results with a sample that is larger in size.

## 5. Conclusions

In summary, we investigated whether and how the degree of response automatization and inter-individual differences in sober fronto-striatal GABA/glutamate levels modulate the effect of acute alcohol intoxication on inhibitory control. We found response automatization to be a prerequisite for finding alcohol-induced inhibition impairments. This was reflected by decreased theta band activity in the left SMA, suggesting this effect to be due to modulations in the encoding of inhibition-enhancing surprise signals. However, we could not predict the degree of alcohol-induced inhibition decline on the basis of sober neurotransmitter levels. This clearly does not imply, however, that alcohol-associated cognitive deficits cannot be induced by alcohol effects on GABAergic and glutamatergic signaling.

## Supplementary Materials

The following are available online at https://www.mdpi.com/2077-0383/9/2/481/s1.
